# Association Between Accumulation of Child Maltreatment and Salivary Oxytocin Level Among Japanese Adolescents

**DOI:** 10.3389/fped.2021.710718

**Published:** 2021-11-29

**Authors:** Rie Mizuki, Takeo Fujiwara

**Affiliations:** ^1^Radiation Medical Science Center for the Fukushima Health Management Survey, Fukushima Medical University, Fukushima, Japan; ^2^Department of Global Health Promotion, Tokyo Medical and Dental University, Tokyo, Japan

**Keywords:** alternative care, physical abuse, emotional neglect, childhood trauma questionnaire, oxytocin

## Abstract

**Background:** Child maltreatment is related to oxytocin (OT), which is related to social functioning. It may hamper the OT level to avoid a harmful situation and increase the OT level to adapt to the situation using a tend-and-befriend stress reaction.

**Objective:** This study aims to examine the association between the accumulation of moderate–severe childhood maltreatment and salivary OT levels in Japanese adolescents.

**Participants:** We used convenience samples of adolescents living in an institution (*n* = 31) and those living with their parents (*n* = 46).

**Methods:** Child maltreatment experiences were measured with the Childhood Trauma Questionnaire. The salivary OT levels were assessed by enzyme linked immunosorbent assay. A multivariate regression analysis was performed to see the association between the accumulation of child maltreatment types and the salivary OT levels adjusted for covariates (i.e., age, sex, and duration of institutionalization).

**Results:** Physical abuse was associated with higher OT, while emotional neglect showed an inverse association with OT. OT was the lowest with one maltreatment type group, which was significantly lower than the non-maltreatment group. As the number of maltreatment types increased from one maltreatment type to 2–3 types and to 4–5 types, OT also increased. This U-shaped association between the number of maltreatment types and OT was confirmed with the significant result of a square term of number of maltreatment type in the model (*p* = 0.012).

**Conclusion:** We found herein a *U*-shaped association between the accumulation of child maltreatment and salivary OT levels. Also, different types of maltreatment had varied effects on the salivary OT. Further study is needed to elucidate the non-linear association between child maltreatment and OT levels.

## Introduction

Child maltreatment is related to a neuropeptide, called oxytocin (OT). Adverse experiences of early life care, such as child abuse and neglect, disrupt the OT system development in children. Early studies on child maltreatment and OT have found negative associations between childhood maltreatment history and OT levels. Heim et al. (1) examined the association between OT levels in the cerebrospinal fluid and the history of moderate–severe childhood maltreatment in healthy adult women. The OT concentrations of women exposed to any type of childhood maltreatment were lower than those without such exposures. A study that investigated children who had been raised in a neglectful orphanage in the first few years of life found their tendency to have lower urinary OT levels compared with controls after interacting with their mothers (2). Similarly, early life stress prior to the age of 13, including maltreatment and loss, was found to reduce plasma OT in healthy adult men (3). Negative associations between childhood emotional maltreatment and plasma OT levels were reported for female adult patients with borderline personality disorder (4) and a population-based adult sample (5). These findings suggest that the OT system development may be hindered by maltreatment experiences during childhood, and peripheral OT could be subsequently maintained at lower levels.

In contrast, several studies indicated differential associations between OT concentrations and maltreatment. Higher reports of maltreatment was positively associated with OT levels in women (6), in a community adult sample raising children (7) and girls with a physical abuse history (8). No difference in the OT levels between maltreated children and controls was also found (9). Such inconsistent findings might be attributed to the heterogeneity in the assessment of the associated childhood maltreatment. Various types of childhood maltreatment (i.e., neglect in general, emotional abuse, emotional neglect, physical abuse, and any maltreatment) have been utilized in research on the OT system. In two studies, maltreatment was not assessed with a self-report instrument and defined by the alternative care placement (i.e., living in a residential care institution or with a foster family). Selecting the type and the definition of maltreatment included in each study seemed rather arbitrary and heterogeneous. Thus, the impact of each maltreatment type on the OT level must be identified. Particularly, multiple forms of maltreatment often happen together, and the detrimental impact of accumulated maltreatment on psychological distress has been brought to societal attention (10–12). In addition to the effect of each maltreatment type on OT, the accumulated experiences of multiple types of maltreatment might differentially affect the OT system.

OT is also linked to social functioning. Enhanced social cognition (13–16) and affiliative behaviors (17–21) were repeatedly reported to be associated with higher OT levels. People with a childhood maltreatment history often suffer from poor social functioning (22, 23), such as disruptive behaviors and relational difficulties from early childhood to adulthood (24–27), as well as impairment with social cognition (28–30). The lower OT levels derived from maltreatment victimization may explain maltreated children's poor social functioning accompanied with the difficulty of developing and maintaining healthy relationships with others. Hence, understanding how experiences of child maltreatment impact the development of OT system may further elucidate the underlying mechanisms of poor social functioning and psychiatric symptoms.

Hence, this study aims to examine the association between the accumulation of moderate–severe childhood maltreatment and the salivary OT levels in Japanese adolescents living in a residential care institution and those living with their parents in the community.

## Materials and Methods

### Participants

Our participants were 31 adolescents (i.e., 19 boys and 12 girls) aged between 13 and 19 years old and living in a residential care institution and 46 adolescents (i.e., 25 boys and 21 girls) in the same age range and living with their two biological parents in the community. A sufficient amount of saliva could not be collected from the two adolescents in the institutionalized group; thus, only 75 adolescents were included in the final analysis. In order to examine the effect of various types of severe maltreatment experiences, it was necessary to approach children who have been institutionalized in residential care mostly due to maltreatment committed by their parents. However, these children, especially adolescents, could be so vulnerable and unstable that obtaining the permissions from residential institutions for research could be usually unfeasible or extremely strenuous. Thus, the current sample was rather rare and valuable. Provided the difficulty associated with recruitment of participants with severe maltreatment experiences, we chose a case-control design and recruited adolescents from the community sufficiently enough to make a comparison with the institutional group.

### Procedure

The participants comprising the institutionalized group were recruited from a residential care institution located in the greater Tokyo area. The research team explained the study to each eligible adolescent at the institution following an agreement with the head of the institution. Although 32 adolescents were approached, and most of them agreed to participate in the study, one child expressed his lack of interest and did not participate, giving us a response rate of 96.9%. The head of the institution signed an informed consent individually for each child, considering each child's emotional and behavioral state.

As for the community sample, snowball sampling, online recruitment, flyers, and word of mouth were used to recruit the participants who were living with their two biological parents. These participants were between the age of 13 and 19 and do not have a history of removal from their family by the Child Guidance Center (similar to the Child Protective Services in the US). Interested parents contacted the research team and provided contact information. Forty-six adolescents from the greater Tokyo area were included in the community group.

For the data collection, a staff of the research team visited the participants' institution or home between 2:00 and 5:00 p.m. These hours were selected to avoid a diurnal fluctuation of the OT values due to food intake, as well as the relative stability of OT in the afternoon and evening hours, as suggested by Forsling et al. (31). The participants were asked not to eat or drink anything other than water 1 h before the researcher's visit. During the data collection session, the researcher first introduced their study and described the consent procedure to the participants. The participants then signed on assent. The saliva samples were collected after a 15 min introduction. Subsequently, the participants filled out a questionnaire packet on the child maltreatment history, mental status, etc., in the environment where the participant can be alone with the researcher. A face sheet, including demographic information, family environment, and involvement with child welfare services, and a questionnaire on the child's behavioral problems were completed by the participant's care worker or a parent.

### Measurements

#### Childhood Maltreatment

The childhood maltreatment the participants experienced was measured with the Japanese version of the Childhood Trauma Questionnaire [CTQ-J; (32)]. The original version of the CTQ is one of the most widely used self-report scales comprising 25 items and assessing the victimization of five types of maltreatment (i.e., emotional abuse, physical abuse, sexual abuse, emotional neglect, and physical neglect) during childhood (33, 34). Each question asks the frequency of the respondent's experience of the indicated event, such as “I had to wear dirty clothes.” The respondents answer questions with a five-point Likert scale: 1 = “never true”; 2 = “rarely true”; 3 = “sometimes true”; 4 = “often true”; and 5 = “very often true.” Five questions compose each type with a score range from five to 25. The severity of each type is defined as “none,” “low,” “moderate,” or “severe” using respective cutoff scores (33). Moderate to severe forms of maltreatment (emotional abuse > 12, physical abuse > 9, sexual abuse > 7, emotional neglect > 14, and physical neglect > 9) were used herein. The internal consistency for the total and each type is as follows: 0.95 for total, 0.87 for emotional abuse, 0.90 for physical abuse, 0.90 for sexual abuse, 0.89 for emotional neglect, and 0.74 for physical neglect. The CTQ-J validity was supported (32).

#### Salivary Oxytocin Level

The saliva samples were collected using Salivatte (Sarstedt, Rommelsdorft, Germany). The participants were asked to chew a roll of cotton for approximately 40 s until it became saturated. Salivates were kept at −20°C and sent to the laboratory, where they were thawed and centrifuged at 4°C at 1,500 × *g* for 15 min. The liquid samples were concentrated by three or four times by lyophilization for a night and kept in −80°C until assayed.

The dry samples were reconstructed with an assay buffer right before the analysis. The assay was performed with a 96-plate commercial oxytocin ELISA kit (Assay Design, MI, USA). The assay procedure was consistent with that in a previous research (35–37). Measurements were performed in duplicate following kit instructions. The optical density of the samples and the oxytocin standards were measured by a microplate reader (Bio-Rad, Richmond, CA, USA) at 405 and 590 nm wavelengths according the relevant standard curves. The intra-assay and inter-assay coefficients were <12.4 and 14.5%, respectively.

### Ethics

We obtained a written assent from each participant before the data collection phase in addition to the written consent from the head of the institution or the parent. Oral approval from their caseworker at the Child Guidance Center was obtained for each participant in the institutionalized group. The institutional review board at the National Center for Child Health and Development approved this study.

### Statistical Analysis

First, Spearman correlation analyses were conducted for the scores of five childhood maltreatment types and demographic variables, including sex, age, and duration of alternative care placement. Second, we categorized the cases into the moderate–severe group for each of the five maltreatment types according to the cutoff scores defined by Bernstein and Fink (33) to test the impact of moderate–severe childhood maltreatment: emotional abuse 13 or more, physical abuse 10 or more, sexual abuse 8 or more, emotional neglect 15 or more, and physical neglect 10 or more. The OT values were log-transformed because their distribution did not meet the assumption of normality. The associations between OT and moderate–severe child maltreatment types were examined with bivariate and multiple regression analyses with and without demographic variable adjustment. Third, multiple regression analyses of OT and the number of maltreatment types were performed to test the cumulative effect of various types of child maltreatment. Multiple regression analysis including a square term was performed to test a quadratic trend, and R squared was compared for goodness of fit for the models with or without a square term. All statistical analyses were conducted with STATA (version 14.2; Stata Corp College Station, TX, United States). The statistical significance level was set at *p* < 0.05 (two-tailed).

## Results

The mean age of the institutionalized group was 15.9 (*SD* = 1.74), and 18 (62.1%) participants were boys. The mean age of the community group was 15.6 (*SD* = 1.84), and 25 (54.4%) participants were boys (Table 1). These two groups did not significantly differ in age and sex. For the institutionalized group, the mean duration of their placement in the current residential care institution was 16.4 months (*SD* = 15.47), ranging from 1 to 59 months. The mean of the total duration of alternative care placement was 4.3 years (*SD* = 5.24), ranging from 1 month to 18 years. Many of these adolescents had lived under alternative care in either another institution or with a foster family before moving to their current institution. On average, they have spent almost one-fourth of their lives separated from their parent/s and family. A child with 18 years in alternative care means that he/she has no experience living with his/her family at home. While no difference in height was observed in total and boys, the girls in the institutionalized group weighed significantly heavier than those in the community group.

**Table 1:** Sample characteristics.

		**Institutionalized** **(***n*** = 29)**			**Community** **(***n*** = 46)**			***P*** **-Test/chi-square**	***p*** **-value**
		***N*** **/Mean**	**%/SD**	**Range**	***N*** **/Mean**	**%/SD**	**Range**		
Age (years)		15.9	1.74	13, 19	15.6	1.84	13, 19	0.67	0.50
Sex	Boys	18	62.1		25	54.4		0.43	0.51
Total duration of alternative care duration (years)		4.3	5.237	0.1, 18					
Current placement duration (months)		16.4	15.47	1, 59					
Height (cm)	All	163.3	8.8	148.5, 176.5	162.3	9.5	140.9, 180	0.44	0.66
	Boys	167.4	7.0	148.5, 176.5	167.7	8.4	140.9, 180	−0.12	0.90
	Girls	156.5	7.2	149.5, 171.5	155.9	6.2	143, 168.4	0.24	0.81
Weight (kg)	All	55.8	9.7	39.2, 77.8	51.9	10.1	35, 82	1.65	0.10
	Boys	58.9	10.8	39.2, 77.8	56.7	10.5	36.8, 82	0.67	0.51
	Girls	50.7	4.4	41, 57.4	46.2	5.8	35, 57	2.25	0.03
**Childhood maltreatment (continuous)**									
Emotional abuse		12.5	6.1	5, 25	6.6	1.9	5, 14	6.16	<0.0001
Physical abuse		13.2	7.1	5, 25	5.5	1.5	5, 13	7.13	<0.0001
Sexual abuse		9.0	6.1	5, 25	5.0	0.3	5, 7	4.37	<0.0001
Emotional neglect		17.6	5.4	5, 25	9.4	3.2	5, 16	8.21	<0.0001
Physical neglect		11.8	4.1	5, 19	6.8	2.4	5, 14	6.67	<0.0001
**Moderate–Severe maltreatment (dichotomous)**									
Emotional abuse		13	44.8		1	2.2		21.31	<0.001
Physical abuse		16	55.2		1	2.2		28.50	<0.001
Sexual abuse		12	41.4		0	0.0		22.66	<0.001
Emotional neglect		23	79.3		3	6.5		41.61	<0.001
Physical neglect		20	69.0		7	15.2		22.30	<0.001
**Number of moderate–severe maltreatment**									
0		2	6.9		36	78.3		49.74	<0.001
1		4	13.8		8	17.4			
2		5	17.2		2	4.4			
3		7	24.1		0	0			
4		6	20.7		0	0			
5		5	17.2		0	0			
Oxytocin (pg/ml)		24.5	10.41	12.6, 51.9	23.8	10.54	10.1, 62.0	0.16^*^	0.87

* *Z for Wilcoxon rank sum test*.

The mean CTQ scores of the five maltreatment types in the institutionalized group were almost double that of the community group (Table 1). The ratio of the adolescents categorized into having moderate–severe maltreatment experiences ranged from 41.4% in sexual abuse to 79.3% in emotional neglect for the institutional group, whereas significantly lower percentages ranging from 0 to 15.2 of the community group reported moderate–severe child maltreatment. Correspondingly, the institutionalized adolescents reported having experienced significantly higher numbers of moderate–severe maltreatment types compared to the community group (χ^2^ = 49.74, *p* < 0.001). More than one in four of the institutionalized group experienced three types of moderate–severe maltreatment and approximately one-third had undergone four or five types. Contrarily, no one in the community group reported having experienced more than two types of moderate–severe maltreatment. No significant difference was found in the raw OT values between the institutionalized (*M* = 24.5 pg/ml; *SD* = 12.57; range: 12.57–51.92) and community (*M* = 23.8 pg/ml; *SD* = 10.54; range 10.10–62.02) groups.

Table 2 presents the results of the Spearman correlation analyses of five types of maltreatment, age, sex, and alternative care duration. The five types of maltreatment showed weak to strong correlations. Strong correlations were observed in emotional abuse with physical abuse (*r*_*s*_ = 0.68, *p* < 0.0001), emotional neglect (*r*_*s*_ = 0.72, *p* < 0.0001), and physical neglect (*r*_*s*_ = 0.60, *p* < 0.0001). Emotional neglect was also strongly correlated with physical neglect (*r*_*s*_ = 0.75, *p* < 0.0001). Moderate correlations were found in physical abuse with emotional neglect (*r*_*s*_ = 0.52, *p* < 0.0001) and physical neglect (*r*_*s*_ = 0.54, *p* < 0.0001), as well as in sexual abuse with physical abuse (*r*_*s*_ = 0.47, *p* < 0.0001), emotional neglect (*r*_*s*_ = 0.47, *p* < 0.0001), and physical neglect (*r*_*s*_ = 0.41, *p* < 0.0005). The correlation between emotional and sexual abuse was weak (*r*_*s*_ = 0.35, *p* < 0.005). The results indicated considerable overlapping occurrences of five different types of maltreatment, particularly among the four maltreatment types other than sexual abuse. The total duration of alternative care was significantly correlated with the five maltreatment types, but not with age or sex, suggesting that alternative care placement was implemented based on adolescents' maltreatment victimization, regardless of their age or sex.

**Table 2:** Spearman correlation coefficients of maltreatment types and demographics.

		**1**	**2**	**3**	**4**	**5**	**6**	**7**
1	Emotional abuse	1.00						
2	Physical abuse	**0.68**	1.00					
3	Sexual abuse	**0.35**	**0.47**	1.00				
4	Emotional neglect	**0.72**	**0.52**	**0.47**	1.00			
5	Physical neglect	**0.60**	**0.54**	**0.41**	**0.75**	1.00		
6	Age	0.02	−0.07	0.15	0.12	0.08	1.00	
7	Sex	0.15	−0.05	0.21	−0.10	−0.07	−0.11	1.00
8	Alternative care duration	**0.48**	**0.62**	**0.59**	**0.64**	**0.55**	0.11	−0.14

*p < 0.05 in bold*.

Table 3 presents the associations between the log-transformed salivary OT concentrations and the five types of moderate–severe maltreatment. None of the bivariate regression analyses between the log-transformed OT values and any of the five maltreatment types resulted in statistically significant. We simultaneously conducted a multiple regression, including the five types, based on the correlations among the five maltreatment types. The results showed that moderate–severe physical abuse (*b* = 0.395, *p* < 0.020) was significantly associated with OT. People who reported a history of physical abuse at moderate to severe levels had significantly higher salivary OT concentrations independent of the effects of the other maltreatment types. The positive association between OT and moderate–severe physical abuse remained significant in the model adjusted for sex and age (*b* = 0.357, *p* = 0.034) and in the model adjusted for sex, age, and alternative care duration (*b* = 0.345, *p* = 0.04). On the contrary, moderate–severe emotional neglect's association with OT was negative and marginally significant in the multiple regression model (*b* = −0.261, *p* = 0.064), as well as in the model adjusted for sex and age (*b* = −0.270, *p* = 0.053). This negative association of moderate–severe emotional neglect reached a statistical significance (*b* = −0.303, *p* = 0.033) when adjusted for sex, age, and alternative care duration. OT was not significantly associated with the other three maltreatment types. These results suggest that child maltreatment may not affect the salivary OT levels in a uniform manner, but would rather derive opposite effects depending on what type of maltreatment a child might have been exposed to.

**Table 3:** Regression coefficients of moderate–severe maltreatment types on oxytocin.

**Moderate–Severe maltreatment type**		**Oxytocin concentration (pg/ml)**		**Bivariate**		**Multiple**		**Sex and age adjust**		**Sex, age, alternative care duration adjusted**	
		**Mean**	***SD***	***B***	***p***	***B***	***p***	***B***	***p***	***B***	***p***
Emotional abuse	(–)	24.0	10.4								
	(+)	24.7	11.1	0.016	0.890	−0.168	0.314	−0.133	0.426	−0.098	0.560
Physical abuse	(–)	23.4	10.5								
	(+)	26.6	10.1	0.138	0.194	**0.395**	0.020	**0.357**	0.034	**0.345**	0.040
Sexual abuse	(–)	24.3	11.1								
	(+)	23.1	6.1	0.0004	0.997	0.014	0.921	0.081	0.562	0.014	0.927
Emotional neglect	(–)	24.3	9.9								
	(+)	23.7	11.5	−0.071	0.450	−0.261	0.064	−0.270	0.053	–**0.303**	0.033
Physical Neglect	(–)	24.1	10.4								
	(+)	24.2	10.6	−0.009	0.923	0.072	0.545	0.057	0.631	0.045	0.705

*p < 0.05 in bold. The oxytocin values in regression analyses were log transformed*.

**Figure 1:**
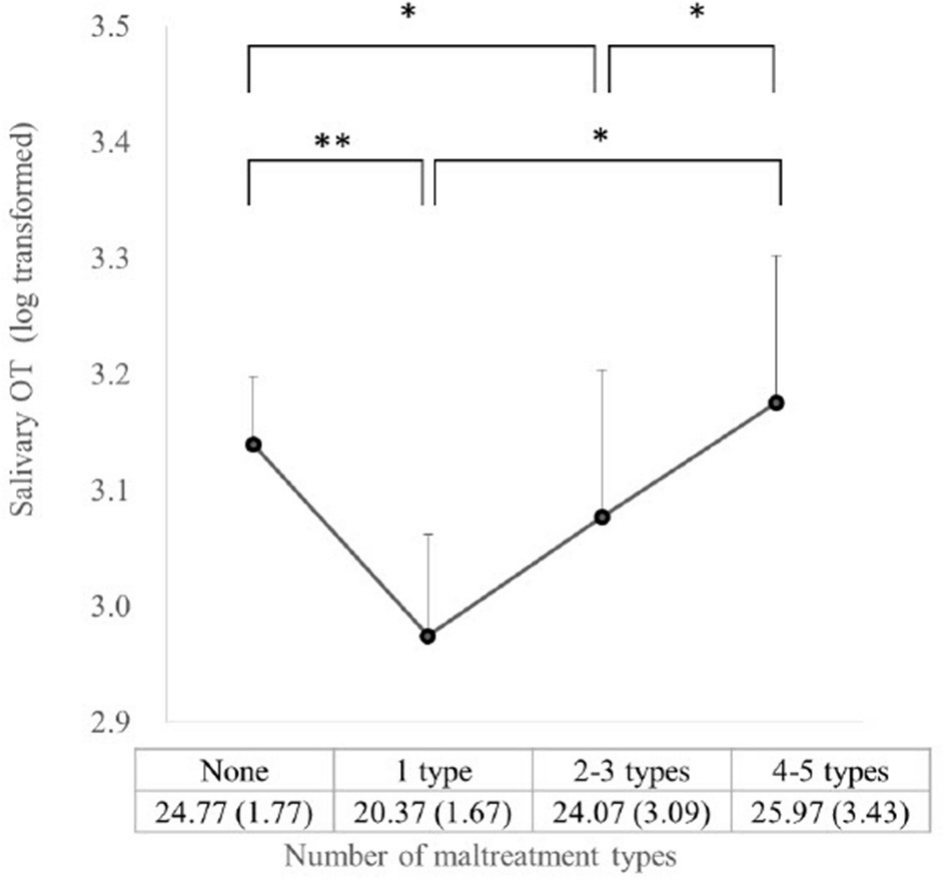
Salivary OT for number of maltreatment types (^*^P < 0.10, ^**^P < 0.05). The mean **(SD)** of the raw OT values for each category of maltreatment type number is presented for graph interpretation.

The cumulative effects of different types of maltreatment on the salivary OT concentration were analyzed using the regression analyses of the number of moderate–severe maltreatment types on the OT concentrations. As in Figure 1, which adjusted for sex, age, and alternative care duration, the group with one type resulted in a statistically significant association (*b* = −0.26, *p* = 0.042), indicating that salivary OT concentration decreased to the lowest, as the number of moderate–severe maltreatment types increased from none to one. When the number of maltreatment types increased to two or three types, the OT concentration slightly increased from that of the group with one type, which was still lower than that of the group without maltreatment experiences (*b* = −0.24, *p* = 0.078). This association was marginally significant. When the maltreatment types reached four or five, the OT level increased and showed no difference from that of the group without maltreatment experiences (*b* = 0.04, *p* = 0.789). However, the OT concentration of the group with four or five types resulted in higher values than either the group with one type (*b* = 0.29, *p* = 0.076) or the group with two or three types (*b* = 0.27, *p* = 0.091), showing an incremental trend in OT as maltreatment type increased from one to four or five. While the trend analysis derived a non-significant result (number of types: *b* = −0.02, *p* = 0.631; *R*^2^ = 0.07) denoting a non-linear relationship, a square term reached statistical significance (number of types: *b* = −0.390, *p* = 0.011; *square term:b* = 0.13, *p* = 0.012; *R*^2^ = 0.15) when added to the multiple regression, indicating a U-shaped association between the number of maltreatment types and OT.

## Discussion

The current study was the first to identify the differential effects of five types of maltreatment on salivary OT concentrations as well as a U-shape pattern of relationship between the accumulation of maltreatment types experienced and OT concentrations in Japanese adolescents. A significant elevation of the OT levels was associated with the presence of moderate–severe physical abuse independent of the effects of the other maltreatment types. On the contrary, moderate–severe emotional neglect significantly decreased the OT levels. Also, regarding the number of maltreatment types, the OT concentrations initially declined to the lowest and then gradually increased as the number of moderate–severe maltreatment types increased. The quadratic relationship between the number of maltreatment types and OT concentrations resulted in statistical significance. The pattern reached the lowest OT levels with one type of maltreatment and increased from one type to two or three types, and to four or five types which reached to the level of no-maltreatment group. The association formed a U-shape.

The higher salivary OT levels among adolescents who experienced moderate–severe physical abuse were consistent with those in the studies reporting higher urinary OT levels in people with a physical abuse history (7, 8). When children suffer from the aggression and violence of their caregivers, they may use a tend-and-befriend response (38). This is a strategy of maintaining social engagement with them to tame down their caregivers' anger instead of escaping from or fighting back against their caregivers (i.e., fight-or-flight response). In this way, children have a chance to consequently avoid being physically hurt without losing the protection and provision from their caregivers. Increased OT levels may facilitate such a response by reducing the reactivity of the cardiovascular system, which allows choosing a response, called a challenge response, that differs from fight-or-flight (39). Higher OT levels may also be attributed to their higher reactivity in the OT system to interpersonal stress. College students who had undergone physical abuse or interpersonal harms exhibited a greater increase in OT levels after social stress compared with those without such experiences (8, 40). OT reduces the activity of the amygdala after exposure to threatening stimuli (41), particularly to social stimuli, such as emotional expression (42). The salivary OT levels may reflect an altered OT system with hyper sensitivity to interpersonal situations due to early-life physical abuse, which can lead to behavioral problems, such as aggression and rejection by peers.

Conversely, adolescents reporting moderate–severe emotional neglect showed lower OT concentrations after controlling the effects of the other maltreatment types. This is consistent with the result of some previous studies with negative associations between OT levels and emotional maltreatment that encompassed emotional neglect and abuse (1, 4, 5). Emotional maltreatment may have a particularly strong damping effect on the OT level (1). The experience of repeated exposure to harsh statements and verbal aggression, as well as the lack of emotional bonding in and support from their family could engender a fearful response of withdrawal of affiliative behaviors, such as children's approaching behaviors toward and communication with their care givers, which is likely to lead to the downregulation of the OT system. This hypothesis is supported by the lack of OT increase in children, who had undergone severe neglect in an orphanage during early life, even after physical contact with their adoptive mothers (2). This could explain the interpersonal difficulty of engaging and maintaining relationships with others, which has often been found in people with a maltreatment history (24, 25, 43). A contradicting finding that higher reports of emotional maltreatment are associated with higher OT levels has been reported in women (6). This study solely focused on emotional neglect and did not examine the effect of the other types of maltreat on OT. Thus, the finding may have resulted from muddling up the up-regulating effect of physical abuse, provided the co-occurrence of four maltreatment types (i.e., emotional and physical abuse and emotional and physical neglect) identified in our findings. Therefore, simultaneously entering different types of maltreatment may be necessary in examining how each type of maltreatment affects the OT system development in children.

The decrease in the salivary OT levels with the number of maltreatment types was inconsistent with the result obtained by Mizuki and Fujiwara (7), who identified a positive linear association between the number of less severe maltreatment types and urinary OT. This may be attributed to the difference in the severity of maltreatment. A less severe form of maltreatment might not have been regarded as a threat and might not have provoked a withdrawal response unlike the type of moderate–severe maltreatment used in this study. In reverse, the decreased OT levels associated with the number of maltreatment types were somewhat similar with those in the previous research of Heim et al. (1), in which a negative relationship was found. Nonetheless, this association showed a linear form and differed from the curvilinear pattern of the current study, in which the OT levels first declined and then gradually increased as the number of experienced maltreatment types increased. This could be attributed to the following reason: people with only one maltreatment type might have experienced emotional maltreatment, which was more frequently experienced (44) and known to suppress salivary OT concentrations, as previously described. Physical abuse would not usually occur as the sole form of maltreatment, but it would concur with emotional maltreatment (45). Cases with one type of maltreatment were less likely to have an experience of physical abuse and would rather have one of the other four types that tend to exhibit a lower OT. Thus, as the count of maltreatment types increased, people were more likely to experience physical abuse, and the effect of physical abuse enhancing the OT concentration would be manifested. The positive effect of physical abuse could eventually surpass the cumulated negative effects of the other types. Consequently, OT and maltreatment formed a U-shaped association.

Furthermore, the genetics associated with the OT system was likely to affect the current findings of the OT levels. The polymorphism of the oxytocin receptor (OXTR), particularly risk alleles, is associated with phenotypic characteristics, such as reduced plasma OT concentrations and poor parenting qualities (46). The OXTR genotypes are regarded as a function of the OT system and show their differential effects according to early life care on social and emotional functioning (47, 48) and salivary OT levels (49). Therefore, the interaction effect between the maltreatment OXTR genes might have partially accounted for the non-linear, *U*-shape association of the current results, provided the high prevalence of the maltreatment history in the current study sample.

The current study has several limitations to address. First, the study participants of both institutionalized and community groups were convenience samples. Particularly, the parents who may have committed moderate-severe abuse are less likely to have their children participate in the study; adolescents with moderate-severe abuse from community may be underrepresented. Careful interpretation and generalization are necessary because the samples did not represent the general population, and the results could be biased by unmeasured confounding factors. Second, the salivary OT analysis process might not be appropriate. While some studies measured the central OT level using the central spinal fluid, the peripheral OT levels were evaluated from the blood, urine, and saliva samples in many others. The use of salivary OT as a proxy of the central OT was suggested by Martin et al. (50) in a study showing modest to strong correlations between salivary and cerebrospinal fluid OT. Meanwhile, the plasma OT was weakly correlated with the central spinal fluid OT. Furthermore, the pre-assay procedure lacking extraction might have affected the results. The comparison between the enzyme immunoassay with and without extraction revealed a much higher plasma OT concentration without extraction (51), implying the possibility of the overestimated OT concentrations of our sample. However, the comparison between the enzyme immunoassay of the samples with and without extraction revealed that the influence of extraction may be much smaller on the salivary sample unlike on the plasma sample (50). The optimal pre-assay procedure still awaits a conclusive evidence. Furthermore, single measurement of salivary OT is pointed as lacking the methodological validity due to instability of OT concentrations within an individual across time (52). Although the stability of peripheral OT levels over 6 months has been reported for parents with infants (37), single adults, and adults who have a romantic partner (53), due to the methodological issues in these studies, the stability peripheral OT is unconvincing. Also, OT system of the adolescent sample in current study is still actively developing which might have been contributing to fluctuation of OT levels. Given uncertainty associated with OT measurements, repeated measurements to detect reliable OT values should be employed to strengthen the validity in the future studies. Third, the OT level could be a result of prenatal exposure to a stressful environment, which could have modified the OT system before exposure to maltreatment in early life and/or social stress derived from the current environment that could enhance the OT levels as coping. These variables must be measured in the future research because we failed to measure them in this study. Fourth, the OT concentrations may reflect the levels of other experiences on the day of the measurement, since OT system is known to be sensitive to interpersonal events, including sexual stimulation, physical contact, or social stress (54–56). The assessment of these social factors will be necessary in future research, to identify the baseline OT levels associated with maltreatment, independent of the effects of social events in the participants' current life. Fifth, the menstrual cycle of female participants were not assessed. Female sex hormones influence the OT system (57); thus, future studies must identify and include the menstrual cycle of female participants into the analysis.

Despite the abovementioned limitations, some noteworthy findings were identified in the current study. The opposite effects of moderate–severe maltreatment on OT depending on the maltreatment type were revealed. Physical abuse increased the salivary OT concentrations, while emotional neglect decreased them. The cumulative effect of the maltreatment types also yielded a U-shaped pattern. The OT levels were the lowest with one type of maltreatment, and OT increased as the number of maltreatment types increased. Further studies need to replicate the current results with a population-based sample and address the effects of genetics, female hormonal cycle, and stresses in the prenatal and current environments to elucidate how childhood maltreatment affects the OT system development in children.

## Data Availability Statement

The original contributions presented in the study are included in the article/supplementary material, further inquiries can be directed to the corresponding author.

## Ethics Statement

The studies involving human participants were reviewed and approved by National Center for Child Health and Development. Written informed consent to participate in this study was provided by the participants' legal guardian/next of kin. Written informed consent was obtained from the minor(s)' legal guardian/next of kin for the publication of any potentially identifiable images or data included in this article.

## Author Contributions

RM and TF contributed to the conception and design of the study and statistical analyses. RM managed data collection and data management, as well as wrote the first draft. TF contributed to critical revision of the manuscript and approved the submitted version.

## Funding

This work was supported by grants from the Research Development Grant for Child Health and Development from the National Center for Child Health and Development (24-12), Ministry of Health, Labour and Welfare (19AA1001), and Grants-in-Aid for Scientific Research from the Japan Society for the Promotion of Science (JSPS KAKENHI Grant No. 21K18294).

## Conflict of Interest

The authors declare that the research was conducted in the absence of any commercial or financial relationships that could be construed as a potential conflict of interest.

## Publisher's Note

All claims expressed in this article are solely those of the authors and do not necessarily represent those of their affiliated organizations, or those of the publisher, the editors and the reviewers. Any product that may be evaluated in this article, or claim that may be made by its manufacturer, is not guaranteed or endorsed by the publisher.
